# Supplementary material for: Investigating the performance of soft robotic adaptive feet with longitudinal and transverse arches
Source: Front Robot AI. 2024 Jul 29;11:1375515. doi: 10.3389/frobt.2024.1375515 (PMC11317230; doi:10.3389/frobt.2024.1375515)
Supplement: Supplementary file 1 [file DataSheet1.pdf]

frontiers

Supplementary Material

SUPPLEMENTARY MATERIALS AND METHODS

Mechanical design of the SoftFoot 3D

As explained in the main text, we introduced some functional, kinematic and implementational *considerations* to decrease the number of combinations resulting from connecting all the links and joints of the five parallel basic 2D modules according to the three connection types (*free*, *elastic*, and *rigid*).

Consideration (C7) about the rigid connection of the midtarsal-ankle joints of the five 2D modules to transmit most of the user's load leads to some consequences. They are listed below:

C7.1 The rear arch and the frontal arch can only pivot on it, so they retain only one rotational DoF (see Fig. S1 (B)).

C7.2 An *elastic* connection on the frontal arch is equivalent to one on the MTP joint, so we consider the frontal arches as elastically connected, and the MTP joints as *free*, for implementation simplicity (see Fig. S1 (C)).

C7.3 Likewise, an *elastic* connection on the rear arches is equivalent to one on the heel joints. Therefore, we connect elastically the rear arches, and let the heel joints be *free* (see Fig. S1 (D)).

C7.4 Similarly, a *rigid* connection on the rear arches is equivalent to one on the heel joints, given that they will anyway behave as rigid body. Therefore, we consider the rear arches as being rigidly connected, while the heel joints as *free* (see Fig. S1 (E)).

C7.5 If the rear arches are rigidly connected, the heel can only rotate around the heel joint, so that it has only one DoF with respect to the adjacent heels (see Fig. S1 (F)).

C7.6 If the heels are rigidly connected, the connection between rear arches and heel joints is indifferent. The rear arch, indeed, can rotate only rigidly around the heel joint with respect to the heel, with a *free* heel joint given considerations C7.3 and C7.4 (see Fig. S1 (G)).

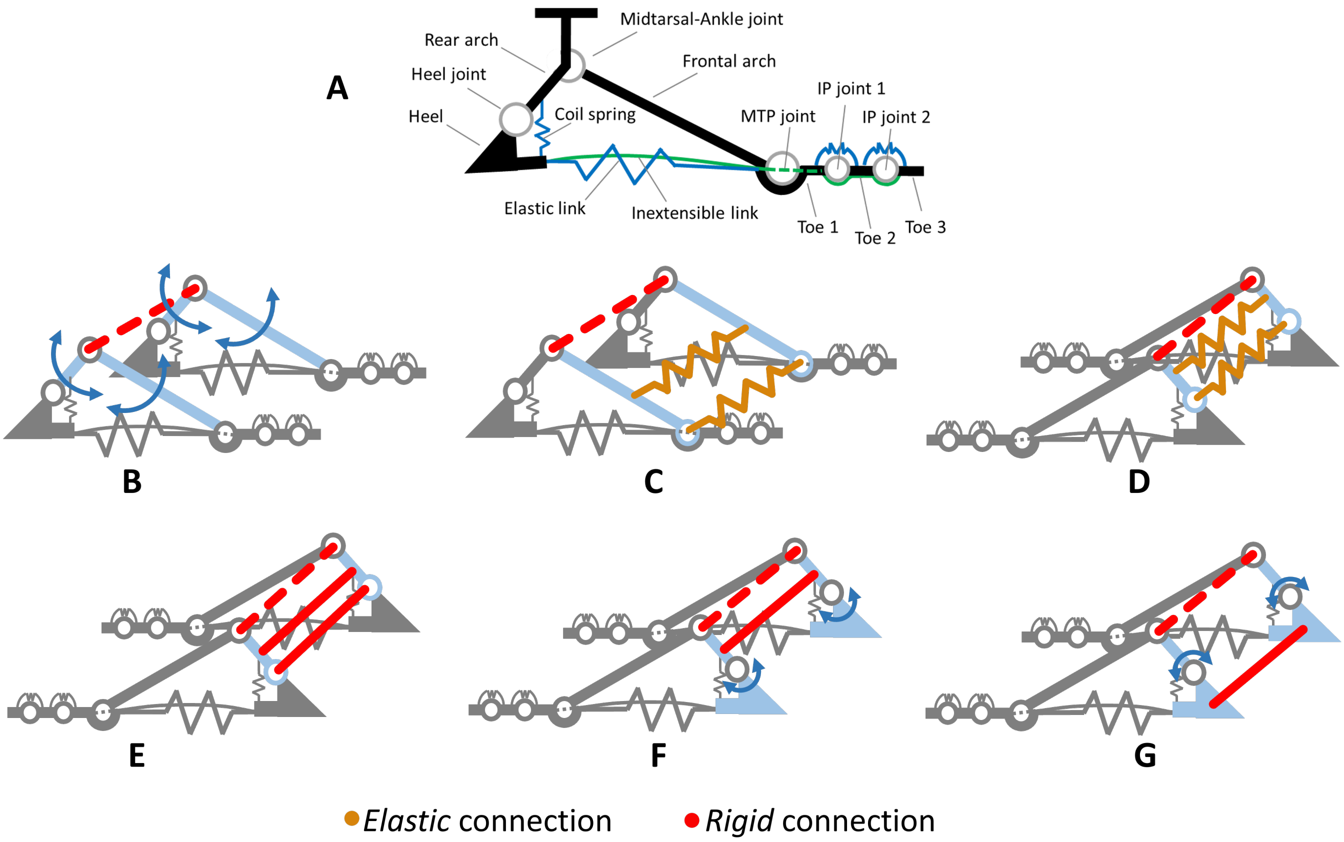

Figure S1. (A) Schematic representation of the six components and the five joints of each 2D module of the SofFoot 3D. (B)-(G) Schematic representation of the considerations deriving from having a rigid connection of the midtarsal-ankle joints of the five 2D modules (represented by the red dashed line). Elastic and rigid connections are respectively shown in orange and in red. Links and joints specifically involved in each consideration are depicted in light blue.
